# Dexamfetamine Use in Pregnancy: Maternal, Neonatal, and Long-Term Neurodevelopmental Outcomes - a Narrative Review

**DOI:** 10.1192/bjo.2026.11854

**Published:** 2026-06-30

**Authors:** Shahnoor Adil

**Affiliations:** Aston Medical School, Birmingham, United Kingdom

## Abstract

**Aims:**

The prevalence of attention deficit/hyperactivity disorder (ADHD) diagnoses and stimulant prescribing among women of reproductive age has increased substantially over the past two decades. Dextro-/amphetamine formulations, particularly dexamphetamine, are now among the most commonly used ADHD medications during pregnancy. Clinical decision making is complicated by uncertainty regarding maternal, fetal and long term child outcomes, leading to high rates of medication discontinuation. This review aims to synthesise current evidence on maternal, neonatal, congenital and long term neurodevelopmental outcomes associated with dexamphetamine and related stimulant use during pregnancy.

**Methods:**

A narrative review of population based cohort studies, registry analyses and clinical guidelines examining perinatal stimulant exposure was conducted. Outcomes of interest included neonatal and obstetric outcomes, congenital malformations, maternal complications and long term neurodevelopmental outcomes, with careful consideration of confounding by maternal ADHD, behavioural and sociodemographic factors. Studies were selected based on their quality, relevance and inclusion of contemporary perinatal data.

**Results:**

Population level data demonstrate a marked rise in perinatal stimulant use, primarily driven by dexamphetamine, with an 11-fold increase observed in a British Columbia registry study (2000-2021). Retrospective cohort studies suggest that continuing dexamphetamine during pregnancy is generally not associated with adverse maternal or neonatal outcomes compared with cessation. Evidence for hypertensive disorders, however, remains inconclusive. International registry studies report no increased risk of major congenital malformations following amphetamine exposure, with a small signal observed for methylphenidate. Long term follow up studies, after adjusting for confounding by maternal ADHD, found no meaningful increase in neurodevelopmental disorders among children exposed in utero. Maternal ADHD and related behavioural or sociodemographic factors may act as confounders underlining the importance of accounting for these factors in research and clinical decision making.

**Conclusion:**

Current evidence suggests that dexamphetamine exposure during pregnancy is not associated with major increases in adverse maternal, neonatal, congenital or long term neurodevelopmental outcomes when confounding is appropriately addressed. Shared, individualised decision making remains essential. Given the limitations of observational study designs and residual confounding, future research should prioritise dexamphetamine-specific data and include qualitative measures of maternal functioning as well as long term childhood outcomes.

